# Prevalence of colorectal symptoms and anal incontinence in patients with pelvic organ prolapse attended at an outpatient urogynecology service

**DOI:** 10.61622/rbgo/2024AO10

**Published:** 2024-03-15

**Authors:** Marco Arellano, Fernanda Santis-Moya, Andrea Maluenda, Alejandro Pattillo, Bernardita Blümel, Dominga Pohlhammer, Silvana Gonzalez, Javier Pizarro-Berdichevsky

**Affiliations:** 1 Corporación de Innovación en Piso Pélvico Hospital Dr. Sótero del Río Santiago Chile Corporación de Innovación en Piso Pélvico, Hospital Dr. Sótero del Río, Santiago, Chile.; 2 Clínica Santa María Santiago Chile Clínica Santa María, Santiago, Chile.; 3 Clínica Puerto Varas Puerto Varas Chile Clínica Puerto Varas, Puerto Varas, Chile.

**Keywords:** Anal incontinence, Colorectal symptoms, Pelvic organ prolapse, Quality of life

## Abstract

**Objective:**

To analyze data of patients with symptomatic pelvic organ prolapse evaluated with PFDI20 and its subscales to report the prevalence of lower gastrointestinal symptoms and anal incontinence in the population of a public hospital and analyze its impact on quality of life.

**Methods:**

Cross-sectional study of patients with symptomatic POP. Patients were evaluated with demographic data, POP-Q, pelvic floor ultrasonography, urological parameters, and pelvic floor symptoms (PFDI-20), and quality of life (P-QoL) surveys. Patients were classified as CRADI-8 "positive" for colorectal symptoms, with responses "moderate" in at least 3 and/or "severe" in at least 2 of the items in the CRADI-8 questionnaires.

**Results:**

One hundred thirteen patients were included. 42.5% (48) were considered positive for colorectal symptoms on CRADI-8. 53.4% presented anal incontinence. No significant differences were found in sociodemographic variables, POP-Q stage, ultrasound parameters, or urological parameters. Positive patients had a significantly worse result in PFDI-20, POPDI (48 vs 28; p<0.001), UDI6 (51 vs 24; p<0.001), and in the areas of social limitation (44.4 vs 22.2; p = 0.045), sleep- energy (61.5 vs 44.4; p = 0.08), and severity (56.8 vs 43.7, p=0.015) according to P-QoL.

**Conclusion:**

Moderate or severe colorectal symptoms are seen in 40% of patients with symptomatic POP in our unit. Full evaluation of pelvic floor dysfunction symptoms should be performed routinely in urogynecology units.

(FONIS SA12I2I53 - NCT02113969).

## Introduction

Pelvic organ prolapse (POP) is a common condition characterized by the herniation of the pelvic organs through the vaginal canal due to the weakening of its support structures.^(1)^ Its estimated prevalence is between 20-50% depending on the definition.^(2)^ The Women’s Health Initiative (WHI) study reports a prevalence of 38-41% of postmenopausal women.^(3)^ The estimated lifetime risk of surgery for pelvic organ prolapse in women is 20.0% by the age of 80.^(4)^ In addition, the evidence shows that the symptoms related to POP greatly affect the quality of life (QoL) in women.^(5)^

Pelvic organ prolapse is part of the Pelvic Floor Dysfunctions (PFD), due to the functional and anatomical relationship of the internal genitalia with the urinary tract and the colorectal canal.^(6)^ Other PFD are urinary incontinence (UI), anal incontinence (AI), obstructed defecation and sexual dysfunction. It has been seen that not only the symptoms of POP, but also the set of symptoms associated with PFD is associated with a significant decrease in quality of life.^(7)^

Colorectal dysfunction includes incontinence and defecation dysfunction such as obstructed defecation. In the United States, a prevalence of anal incontinence of 6-28% has been reported.^(8)^ An epidemiological association has been established between different PFD, even sharing risk factors such as parity or pelvic floor surgeries.^(9,10)^ However, there is little information regarding the coexistence of colorectal symptoms in patients with POP^(11–14)^ and its impact on quality of life.

There is evidence that these symptoms are not reported spontaneously by patients, so they are generally underestimated.^(10)^ On the other hand, specialists do not always investigate colorectal symptoms, despite having standardized and validated instruments for their evaluation such as the PFDI-20.

The purpose of our study is to describe the prevalence of colorectal symptoms through validated scales and subscales in patients with symptomatic POP who seek treatment, and to analyze their impact on QoL.

## Methods

This study was approved by the Institutional Ethics Committee of Hospital Sótero del Río and was carried out in accordance with its recommendations.

In our center, in 2013, a cross-sectional sub-analysis of patients being followed up by a prospective study on the use of pessaries for the management of POP was performed, which included the standardized application of the PFDI-20 questionnaire with its subscales (POPDI, UDI6 and CRADI-8). The patients were recruited between September 2013 and May 2014 at the Centro de Innovación en Piso Pélvico (CIPP) of the Hospital Sótero del Río.

All women, regardless of age, who were referred to the CIPP due to symptomatic POP in the described period and who agreed to participate in the study were included. Patients with incomplete questionnaires or without POP-Q were excluded.

Upon admission, demographic data, clinical parameters (prolapse stage, clinical symptoms, associated quality of life alteration, and sexual impact of POP), 2-hour Pad test, 300cc stress test, and translabial pelvic ultrasound were obtained. A 3-day voiding diary was carried out prior to the next consultation. POP stage was assessed with POP-Q.

Various scales and questionnaires have been designed to assess these symptoms.^(15)^ The PFDI-20 (Pelvic Floor Distress Inventory-20) is an instrument designed to measure symptoms and impact on quality of life in patients with PFD.^(16)^ The PFDI-20 is made up of 3 subscales: UDI-6 (Urinary Distress Inventory - 6), POPDI-6 (Pelvic Organ Prolapse Distress Inventory - 6) and CRADI-8 (Colorectal-Anal Distress Inventory - 8). Each of the scales evaluates from 0 to 100 the level of impact of these pathologies on the patients. The PFDI-20 score is the sum of the 3 subscales. This makes it useful for the simultaneous assessment of symptoms in patients with POP.

In the study, quality of life was evaluated with a version validated for the Chilean population of PFDI-20.^(16)^ PFD symptoms were evaluated with the version adapted to the Chilean population of PFDI-20.^(15)^ Sexually active patients were evaluated with the version adapted to Chile of PISQ-12 (12-item Pelvic Organ Prolapse/Urinary Incontinence Sexual Questionnaire).^(17)^

The CRADI-8 scale was used to identify patients with significant colorectal symptoms. In this study, we considered CRADI-8 positive for colorectal symptoms in cases in which the response to symptoms was "moderate" impact in at least 3 and/or "severe" in at least 2 of the 8 items of this subscale, corresponding to a minimum value of 25 points out of a possible total of 100. In addition, the prevalence of anal incontinence was analyzed independently. The decision for this cut-off point was discretional, based on the expert panel of our pelvic floor group in the hospital, of what we considered could be highly sensitive for high bothersome colorectal symptoms. Anal incontinence was defined and based on questions 3, 4, and 5 of CRADI-8.

SPSS was used for data analysis. T-student or U-Mann-Whitney test was used for continuous variables, and the Chi-square test or Fisher’s exact test for categorical variables. Results with a p<0.05 value were considered significant. Logistic regression aiming to predict CRADI-positive patients was performed including age, parity, BMI, age at menopause, history of hysterectomy, use of hormone replacement therapy, history of forceps, stage of prolapse.

## Results

One hundred sixteen patients were recruited. Of these, 3 patients with incomplete surveys were excluded, so 113 patients were considered for the final analysis. Of these patients, 48 (42.5%) met the criteria to be considered CRADI-8 positive. The general characteristics of the population are shown in table 1. The average age was 64.4 ± 8.6 years, the average parity was 3.4 ± 1.8, and body mass index (BMI) was 29.4 ± 4.5 kg/m2. No significant differences were found between the general characteristics when comparing the CRADI positive and CRADI negative groups (Age, parity, BMI, age at menopause, history of hysterectomy, use of hormone replacement therapy, history of forceps).

**Table 1:** General and specific demographic characteristics

	CRADI (-) Average ± SD	CRADI (+) Average ± SD	p value
Age	65 ± 7.3	63.7 ± 10	0.421
BMI	29.7 ± 4.1	28.9 ± 4.9	0.372
Parity	3.2 ± 1.4	3.8 ± 2.3	0.296
Heavier newborn weight	3679.2 ± 929.5	3661.0 ± 559.1	0.864
Age in menopause	47.7 ± 5.7	46.9 ± 4.7	0.316
	**CRADI (-)** **n(%)**	**CRADI (+) n(%)**	**p value**
Smoking	12(18.5)	11(23.4)	0.342
Menopause	60(92.3)	44(93.6)	0.549
Forceps	16(24.6)	17(36.2)	0.133
Hormone replacement therapy	7(10.8)	2(4.3)	0.186
Hysterectomy	8(12.3)	6(12.8)	0.581
Previous prolapse surgery	4(6.2)	1(2.1)	0.299

Anal incontinence corresponding to questions 3, 4 and 5 of the CRADI-8 was specifically evaluated. It was found that 62 patients (53.4%) presented anal incontinence, of which 54 patients (46.5%) presented flatal incontinence, and 27 patients (23.2%) presented fecal incontinence. The patients were compared regarding the objective evaluation of PFD elements (POP and urological). No differences were found between both groups in POP-Q stage, POP ultrasound parameters, post-void residue, voiding diary, pad test or stress test with 300 ml and POP reduction. The symptoms of PFD were evaluated through the PFDI-20 and subscales, shown in table 2. It was found that the CRADI-8 positive patients also had worse results in urinary incontinence by UDI-6 (51 vs 24; p<0.001). In addition, CRADI-8 positive patients had significantly worse performance in POP symptoms measured by POPDI (48*vs* 28; p<0.001) and overall by PFDI-20.

**Table 2:** Specific characteristics

	CRADI (-)	CRADI (+)
POP-Q Stage I	14(21.5)	14(29.2)
POP-Q Stage II	44(67.7)	26(54.2)
POP-Q Stage III	7(10.8)	8(16.7)
POP-Q Stage IV	51(78.5)	34(70.8)
Positive PAD Test	14(23.3)	9(20.9)
Positive Stress test	41(68.3)	34(70.8)

	CRADI (-) Median (IQR)	CRADI (+) Median (IQR)	p-value
PDFIT	50 (0 - 50)	138 (75 - 188)	< 0.001
POPDI	25 (0 - 50)	50 (25 - 75)	< 0.001
UDIT	25 (0 - 50)	50 (25 - 75)	< 0.001
CRADIT	0	25 (25 - 50)	< 0.001

	CRADI (-) Average ± SD	CRADI (+) Average ± SD	p value
Voiding diary: Pads	0.8 ± 1.7	1.7 ± 2.9	0.067
Voiding diary: stress incontinence episodes	2.2 ± 4.1	2.6 ± 3.7	0.595
Voiding diary: urge incontinence	1.4 ± 3.4	2.1 ± 3.2	0.296
Voiding diary: urgency	2.0 ± 3.8	3.2 ± 4.0	0.296
Ultrasonographic PVR	54.86 ± 71.39	58.78 ± 73.74	0.778
Catheterization PVR	32 ± 53	25 ± 36	0,417

PVR – post void residue

Finally, the impact of PFD symptoms on quality of life was evaluated using the P-QoL reported in table 3. In general, CRADI-8 positive patients had significantly worse results (75 vs 50; p < 0.001). In addition, they performed significantly worse in social limitation (44.4 vs 22.2; p = 0.045), sleep and energy (61.5 vs 44.4; p = 0.08) and severity (56.8 vs 43.7, p=0.015). The differences in role limitation (83.3 vs 66.7; p = 0.064), physical limitation (83.3 vs 66.7, p = 0.062) and emotions (72.2 vs 55.6, p= 0.071) did not reach statistical significant differences.

**Table 3:** Comparison QoL, social limitation, severity, sleep-energy

	CRADI (-) Median (IQR)	CRADI (+) Median (IQR)	p-value
PQoL General Health Perceptions	50 (25 - 75)	75 (50 - 75)	< 0.001
PQoL Prolapse Impact	66,7 (66,7 - 100)	84 (66,7 - 100)	0.092
PQoL Role limitations	66,7 (33,3 - 100)	83,3 (50 - 100)	0.064
PQoL Physical limitations	66,7 (16,7 - 100)	83,3 (50 - 100)	0.062
PQoL Social limitations	22,2 (0,0 - 66,7)	44,4 (11.1 - 88,9)	0.045
PQoL Personal Relationships	0 (0 - 66,7)	0 (0 - 66,7)	0.742
PQoL Emotions	55,6 (22,2 - 88,9)	72,2 (33,3 - 100)	0.71
PQoL Sleep/Energy	33,3 (16,7 - 66,7)	66,7 (33,3 - 100)	0.008
PQoL Severity Measures	41,7 (25 - 58,3)	58,3 (33,3 - 83,3)	0.15

	CRADI (-) Average ± SD	CRADI (+) Average ± SD	p-value
PQoL General Health Perceptions	51.2 ± 23.6	67.7 ± 21.9	< 0.001
PQoL Prolapse Impact	74.9 ± 28.9	84 ± 27.5	0.092
PQoL Role limitations	60.1 ± 36.7	72.6 ± 32.3	0.064
PQoL Physical limitations	57.7 ± 38.8	70.8 ± 33.6	0.062
PQoL Social limitations	33.2 ± 35.3	47.2 ± 88.9	0.045
PQoL Personal Relationships	31.8 ± 41.3	34.4 ± 41.2	0.742
PQoL Emotions	55 ± 36	67.1 ± 33.2	0.71
PQoL Sleep/Energy	44.4 ± 31.9	61.5 ± 34.6	0.08
PQoL Severity Measures	43.7 ± 28.4	56.8 ± 26.6	0.15

Logistic regression was performed among CRADI-positive and CRADI-negative patients, and there were no significant differences among them (Table 4). We did a post hoc power calculation and calculated a 91% power (incidence in population 28%, incidence in study group 42.5%, for 113 subjects, and an alpha error of 0.05).

**Table 4:** Multivariate analysis

Variables	OR	95% CI	p-value
Age	0.987	0.935-1.042	0.634
Previous POP surgery	0.310	0.031-3.098	0.319
Previous hysterectomy	0.987	0.298-3.266	0.983
Smoking	1.268	0.449-3.579	0.654
Menopause	1.835	0.350-9.615	0.473
Forceps	1.652	0.715-3.815	0.240
Stage III/IV POP	0.666	0.264-1.678	0.388

## Discussion

Moderate or severe colorectal symptoms affect 40% of the patients with symptomatic POP in our section. These findings are also associated with a greater presence of urinary symptoms and POP, which in turn translate into a poorer quality of life.

A study in 2010 by Ha et al.^(18)^ evaluated 265 women to determine the prevalence of colorectal symptoms and fecal incontinence in patients with UI and POP in a Latino population using the CRADI-8. 42.3% of the patients presented at least one colorectal symptom that was quite bothersome, with a prevalence of fecal incontinence of 58%. Another study carried out in Brazil by Portella et al. demonstrated a 40.54% prevalence of FI in patients with UI, and 27.91% in patients with POP.^(19)^ This is in concordance with what was found in our study.

In Chile, no studies have been carried out specifically in the population with POP. In 2016 a survey study was carried out at the Hospital Clínico de la Universidad de Chile including 1136 patients who attended a clinical center for various pathologies, excluding those who attended for coloproctological or gynecological pathology, of which 59.2% were women. Of the women, 32.8% of the patients presented fecal incontinence.^(20)^ These results show how in the general population, there is a high percentage of women who suffer from colorectal symptoms, which reinforces the importance of conducting a targeted evaluation. It would be desirable to carry out a study in the general population to assess not only the prevalence of fecal incontinence, but also the presence of any colorectal symptoms and their association with PFD.

A study by Kahn et al.,^(21)^ found significant differences in colorectal symptoms (anal incontinence, constipation) with different stages of prolapse (0-IV), however, when verified by multivariate analysis, as in our study, these differences were not significant. Patients can also present colorectal symptoms that affect quality of life regardless of the stage of prolapse. Also, symptoms may be related to functional disorders, more than anatomical disorders.

Saks et al.^(11)^ found significant differences by multivariate analysis, with a higher prevalence of intestinal obstructive symptoms in patients with posterior wall prolapse, but there was no relationship between the severity of these symptoms and the severity of posterior prolapse. These findings persist once confounding variables such as BMI, age, among others, were corrected.

Bezerra et al.^(10)^ found that 54.6% of the women who consulted for PFD in the urogynecology unit had defecation disorders, of which 67% were constipation, 41.1% fecal incontinence, and 34% defecation urgency. These findings were significantly higher in patients with PFD and considerably affected the quality of life,^(10)^ which highlights the need to better describe this population and evaluate these symptoms in a targeted manner.

Due to the significant percentage of women who present these symptoms classified as moderate and severe in our study, we believe that a specific evaluation of all possible symptoms of pelvic floor dysfunction should be carried out, independent of the primary reason for consultation, in the urogynecology and general consultation units. In this way, we can offer a treatment or counseling plan that includes all areas of your pathology, regardless of the primary reason for consultation, improving the clinical experience and general satisfaction of our users.

A strength of this study is the use of standardized questionnaires to measure the presence and severity of symptoms, which constitutes a fundamental tool to compare between populations and to collaborate in the study of these alterations in other countries. Another strength is that the data collection was in the context of a prospective study, which reduces data collection bias.

Another of the strengths is that little is known in the Latin American population about the prevalence of these symptoms in patients with prolapse, so we believe that it is a contribution to the way medicine is practiced Latin America. This would make it possible to offer a comprehensive, multidisciplinary management plan that solves in the widest possible way the recovery of an adequate state of health with a greater impact on improving the quality of life of our patients.

Among the weaknesses is, as already mentioned, that it is a very selective population of patients with POP, which may not be representative of the general population, but of the population to be evaluated in a urogynecology and pelvic floor polyclinic. Another weakness is that, being a cross-sectional study, cause and effect cannot be determined, so it is not possible to determine if it is pelvic organ prolapse that causes colorectal symptoms or vice versa.

## Conclusion

It is essential to understand pelvic floor problems in a comprehensive way to be able to help patients in an interdisciplinary way and thus contribute to improving their quality of life. Therefore, there would be an association between colorectal problems such as constipation, obstructed defecation, fecal incontinence, urinary incontinence and POP, understanding a common underlying pathophysiology. However, more studies are needed that include patients without genital prolapse, to assess the real relationship between colorectal symptoms and POP and thus make more comprehensive decisions, such as planning surgical or conservative treatment, and thus help in the definitive rehabilitation of our patients.
